# A Prospective Multicenter Luminex-Based Clinical Algorithm to Define Unacceptable HLA Mismatches Before Kidney Transplantation. Consequences on Outcome, Waiting Time, and Wait List Composition

**DOI:** 10.3389/ti.2025.15497

**Published:** 2026-01-14

**Authors:** Fabian Köppen, Martina Koch, Kai Lopau, Katharina Heller, Markus Luber, Bernd Spriewald, Kerstin Amann, Achim Jung, Julia Weinmann-Menke, Thomas Drasch, Jens Werner, Bernhard Banas, Daniel Zecher

**Affiliations:** 1 Department of Nephrology, Regensburg University Hospital, Regensburg, Germany; 2 Department of General, Visceral and Transplantation Surgery, University Medical Center, Mainz, Germany; 3 Department of Internal Medicine, University of Würzburg, Würzburg, Germany; 4 Department of Nephrology and Hypertension, University Hospital Erlangen, Erlangen, Germany; 5 Department of Internal Medicine 5-Hematology and Oncology, University Hospital Erlangen, Erlangen, Germany; 6 Department of Nephropathology, Institute of Pathology, University of Erlangen-Nürnberg, Erlangen, Germany; 7 Institute of Transfusion Medicine -Transfusion Centre, Johannes Gutenberg University Medical Centre, Mainz, Germany; 8 1st Department of Medicine, Division of Nephrology, Mainz University Hospital, Mainz, Germany; 9 Department of Surgery, Regensburg University Hospital, Regensburg, Germany

**Keywords:** highly sensitized, kidney transplantation (KT), outcome, unacceptable HLA antigen mismatches, waiting time

## Abstract

Determination of unacceptable human leukocyte antigen (HLA) mismatches (UAM) before kidney transplantation (KT) aims at minimizing immunological risk and routinely involves Luminex single antigen bead (SAB) testing. SAB-UAM criteria, however, often lack standardization. We implemented standardized mean fluorescence intensity (MFI)-based SAB-UAM criteria in four German transplant centers and prospectively studied the consequences on waitlist composition as well as waiting time, early antibody-mediated rejection (AMR) and graft loss in 267 patients. HLA were deemed unacceptable in case of CDC-reactivity or antibodies against known HLA from previous transplants irrespective of MFI. For all other antibodies, the MFI cut-off was 5.000 with the exception of 10.000 for anti-HLA DQ. We observed significant accumulation of highly sensitized patients (virtual panel-reactivity >95%) on the waiting list during the study period. Median time to KT was longer in patients with UAM, but differences were not statistically significant. Patients with preformed donor-specific anti-HLA antibodies (DSA) below the UAM cut-off criteria (39/267) experienced more AMR episodes compared to DSA-negative patients (10.3% vs. 1.3%, p < 0.001). Graft survival, however, was not statistically different over a median follow-up of four years. Standardized SAB-UAM criteria associated with good short-term outcomes but resulted in accumulation of highly sensitized patients on the waiting list.

## Graphical Abstract

**Figure:**
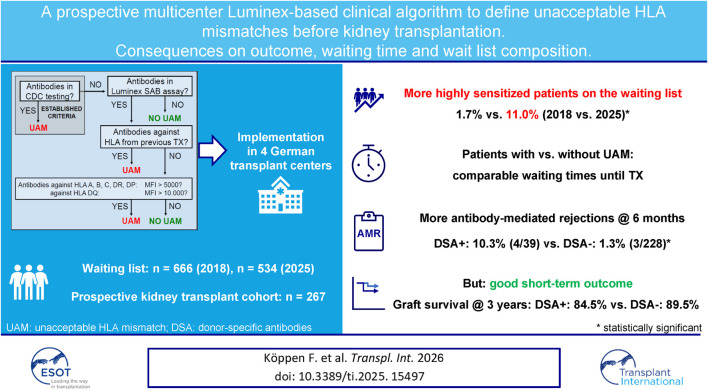

## Introduction

Successful kidney transplantation (KT) remains a cornerstone in the treatment of end-stage renal disease [1], significantly improving patient survival and quality of life [2, 3]. Overcoming the immunological barriers between donor and recipient, however, remains a critical challenge.

To avoid transplantation of human leukocyte antigen (HLA)-incompatible grafts with a high risk of early antibody-mediated rejection (AMR) and premature graft loss, transplant physicians and tissue typing laboratories have for long defined unacceptable HLA antigen mismatches (UAM) prior to KT. When a patient has anti-HLA antibodies that are considered high-risk, organs carrying these HLA will be excluded for a patient and the respective HLA will be declared unacceptable. The stricter UAM are defined, the lower is the risk of early rejection at the cost of prolonging waiting times due to an increasing donor pool restriction [4–6].

In the last 20 years, the Luminex single antigen bead (SAB) test has revolutionized anti-HLA antibody detection, providing a highly sensitive and specific semiquantitative measurement of antibody strength expressed as mean fluorescence intensity (MFI). Many studies have demonstrated that the presence of donor-specific anti-HLA antibodies (DSA) detected by the SAB test prior to KT correlates with an increased risk of early AMR and graft loss, even in the absence of cytotoxicity in CDC assays [7–12].

The relationship between the MFI and clinical outcomes in DSA-positive patients is less clear [13]. Whereas some studies have demonstrated a positive association between MFI levels and the incidence of early AMR and premature graft loss [7, 8, 10, 14–16], other studies have reported poorer graft survival in DSA-positive patients regardless of MFI levels [10, 12, 17].

The SAB test has some well-described technical limitations that can result in false-positive results [17, 18]. Moreover, the lack of a truly quantitative measure and potential differences in pathogenicity do not allow for a precise prediction of the immunological risk of a given antibody based on its MFI alone, resulting in a low predictive value of DSA in an individual patient [19]. Consequently, UAM algorithms are almost always individualized, lack standardization, and are highly variable between transplant centers.

In an attempt to standardize UAM criteria and balance the risk between early immunological complications and prolonged waiting times, we implemented CDC- and MFI-based SAB-UAM criteria at four German transplant centers. We used MFI thresholds that had previously been shown to result in excellent short-term clinical outcomes [20]. A retrospective analysis applying the same SAB-UAM criteria to a cohort transplanted at our own center in the pre-Luminex era further suggested that patients transplanted against DSA that fulfilled these SAB-UAM criteria had a high risk of premature graft loss, whereas patients with preformed DSA below the thresholds of our algorithm had excellent outcomes [19]. To further minimize risk, all known HLA from previous transplants were deemed unacceptable if antibodies against these HLA were detected in the SAB test [21, 22]. In this manuscript, we give a comprehensive overview of the consequences of this SAB-UAM algorithm, namely changes in waitlist composition over time as well as the impact on waiting time prior to KT, the incidence of early AMR, and graft loss, in a prospective cohort of KT patients.

## Patients and Methods

### HLA Typing

Serological HLA typing of both donors and recipients was performed according to standards of the European Federation for Immunogenetics. During patient recruitment (01.01.2019 until 31.12.2021), donor and recipient HLA typing was only mandatory for HLA-A, -B, and -DR in the Eurotransplant region but was most often extended by the local tissue typing laboratories. Completeness of 11-loci donor and recipient HLA typing is shown in Supplementary Table S1.

### HLA Antibody Testing

For three transplant centers (Regensburg, GRBTP; Würzburg, GWZTP; and Erlangen, GNBTP), HLA antibody testing was done at quarterly intervals in the tissue typing laboratory at Erlangen University Hospital. Screening was done using a commercial solid-phase microsphere-based assay (LSM12; One Lambda Inc., Los Angeles, CA). Sera were analyzed on a LABScan 200 Luminex (Luminex Corp., Austin, TX) flow analyzer, applying a threshold ratio for positive results of 2.5. In positive sera, HLA specificity was determined by a single-antigen assay for HLA class I and/or HLA class II antigens (LABScreen Single Antigen, Class I or II, respectively, both One Lambda Inc.). The tests were performed according to the manufacturers’ instructions and analyzed on a LABScan 200 Luminex flow analyzer, applying a baseline-adjusted MFI cutoff for positive reactions of 500.

In Mainz (GMZTP), screening and specification of HLA antibodies was performed using a commercial solid-phase microsphere-based assay (LSA Class I and Class II; Immucor GTI Diagnostics Inc., Waukesha, WI, USA). Sera were analyzed on a LABScan 200 Luminex flow analyzer (Luminex Corp., Austin, TX). All assays were conducted according to the manufacturers’ instructions. Sera were considered positive for specific HLA antibodies when the raw MFI was above 750 and the MFI/LRA (lowest ranked antigen) ratio was greater than the bead/lot-specific cut-off provided by the manufacturer.

### Definition of Luminex-Based Unacceptable HLA Antigen Mismatches (SAB-UAM)

**FIGURE 1:**
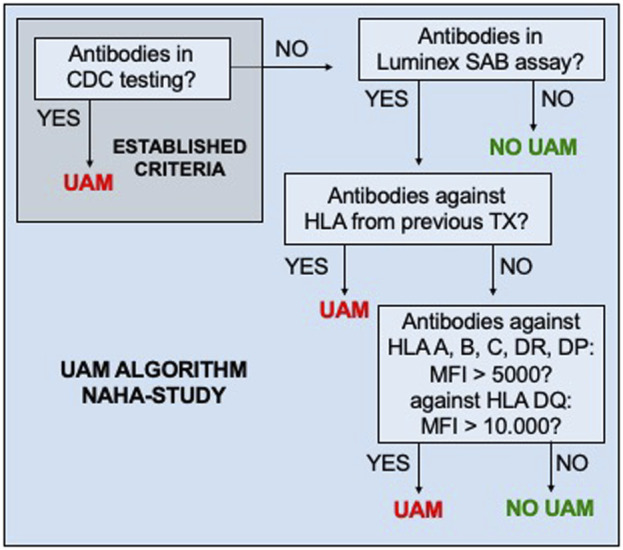
Standardized criteria of unacceptable HLA mismatches (UAM). CDC, complement-dependent cytotoxicity; MFI, mean fluorescence intensity of anti-HLA antibodies detected in the Luminex single-antigen bead (SAB) test.

HLA were classified as UAM prior to KT if at least one of the predefined criteria (Figure 1) were met at any time. Once an HLA was classified as unacceptable, it remained listed as such, irrespective of subsequent reductions in antibody MFI, lack of antibody detection, or a negative result in CDC-testing. During patient recruitment, UAM could only be reported to ET on the serological level.

### Impact of SAB-UAM Assignment on Wait List Composition and Waiting Time

The impact of UAM on waiting time in adult (≥18 years) patients listed for KT via the standard Eurotransplant Kidney Allocation System (ETKAS) or the Eurotransplant Senior Program (ESP) was studied in a cross-sectional approach at five time points. The first was in September 2018, prior to implementation of the current SAB-UAM algorithm. Until then, UAM assignment was not systematically performed but rather done on an individual patient’s basis, considering mostly CDC-specificities and HLA against which antibodies directed against HLA from previous transplants were detected in Luminex SAB testing. The remaining time points were after implementation of the current SAB-UAM algorithm at the three transplant centers (GRBTP, GWZTP, and GNBTP) in June 2019, and three (March 2022), four (May 2023) and six (June 2025) years later. As in GMZTP, the SAB-UAM criteria were only implemented in February 2020, the GMZTP June 2019 waitlist data were omitted from analysis. Highly immunized patients listed in the acceptable mismatch (AM)-program were excluded, as were patients listed for multi-organ transplantation, with kidney-after-other-organ status, or with a high urgency status. Waiting time was defined as the time between the date of first dialysis and the respective reference date. Virtual panel reactivity (vPRA) levels were calculated based on UAM by ET using the Eurotransplant Reference Laboratory (ETRL) donor frequency calculator at https://www.etrl.org (last accessed on June 25, 2025).

### Clinical Study Protocol and Patients

The SAB-UAM algorithm was prospectively implemented for all adult (≥18 years) patients on the kidney and kidney-pancreas waiting lists of the participating transplant centers (GRBTP starting 01.01.2019, GWZTP on 21.02.2019, GNBTP on 01.05.2019, and GMZTP on 01.02.2020) and was maintained unchanged until the end of the recruitment phase on 31.12.2021. Patient recruitment into the study, however, varied considerably between the four centers, mostly because of the constraints of the COVID pandemic in GNBTP and GWZTP (Supplementary Figure S1).

Patients that were transplanted against UAM for any reason but included in the study were excluded from analyses (n = 4). Study data were collected using REDCap electronic data capture tools hosted at Regensburg University Hospital [23] at baseline (day of KT) as well as at 14 days, 3 months, 6 months, 12 months, and then yearly thereafter.

All patients gave their written informed consent. The study was approved by the local institutional review boards of the participating centers (GRBTP 18-1153_1-101, GNBTP 410_19 Bc, GWUTP 9/19_awbz, and GMZTP 2019-14663_1-NIS).

### Assignment of DSA

Patients were categorized as DSA-positive if they had HLA antibodies against donor HLA in the most recent Luminex SAB assay prior to transplantation. Assignment of donor-specificity was performed on the serological level based on the available donor HLA typing. In cases of DSA against self-HLA, high-resolution typing of both donor and recipient was performed retrospectively (n = 4). This approach revealed true donor-specificity in 1/4 cases. All other cases were counted as DSA-negative. Missing HLA typing was retrospectively performed in case of potential DSA. With this approach, patient categorization into DSA-positive or DSA-negative was possible in all patients with detectable anti-HLA antibodies. DSA were considered positive with MFI ≥1000 in the most recent SAB assay prior to KT.

### Diagnosis of Rejection

All rejection episodes were biopsy-proven. Biopsies were obtained either as protocol biopsies on days 14, 90, and at 1 year (GRBTP) or when clinically indicated (all centers). Specimens were evaluated on light microscopy and immunohistochemistry for C4d and SV40 staining and were graded according to the BANFF 2019 classification [24].

### Statistical Analysis

Statistical analysis was performed using IBM SPSS version 28.0.0.0 (SPSS Inc., Chicago, IL, USA). Data are presented as median (interquartile range, IQR) or median (range). For categorial data, comparisons were based on the chi-square test or Fisher’s exact test. Mann-Whitney-U- and Kruskal-Wallis-tests were used to compare interval scaled or metric data. The Kaplan-Meier method was used to conduct survival analyses and group differences were evaluated by the log-rank test. All tests performed were two-sided. P < 0.05 was considered statistically significant.

## Results

### Consequences of SAB-UAM on Wait List Composition and Waiting Time

We first analyzed the consequences of the new SAB-UAM algorithm on the waiting list composition of the four participating transplant centers after exclusion of all highly sensitized patients listed in the AM program. Cross-sectional analysis of the active kidney waiting list at various time points over a period of 7 years revealed a continuous decrease from 666 patients in 2018 to 534 patients in 2025 (Table 1), following a general trend in Germany [25]. Implementation of SAB-UAM in early 2019 in three of the participating centers resulted in a fourfold increase in patients with vPRA > 95% (1.7% vs. 7.3%, p < 0.001). Median vPRA in sensitized patients also increased significantly from 60.4% to 81.5% (p < 0.001) (Table 1). Ever since, the proportion of sensitized patients (vPRA >0%) continuously increased from 19.8% in 2018 to 39.3% in 2025, with the most dramatic effect on the proportion of patients with vPRA >95% (1.7% vs. 11.0%, p < 0.001) (Table 1). Whereas overall waiting time did not change significantly over time (Supplementary Table S2), we noticed accumulation of highly sensitized patients (vPRA >95%) who waited 3 years longer in 2025 as compared to non-sensitized patients (7.3 vs. 4.2 years, p < 0.001, Table 2).

**TABLE 1:** vPRA over time in patients on the waiting list.

vPRA category	Time of analysis					
	09/2018 n = 666	06/2019^a^ n = 590	03/2022 n = 622	05/2023 n = 563	06/2025 n = 534	*p*
vPRA = 0%	534 (80.2)	442 (74.9)	424 (68.2)	369 (65.5)	324 (60.7)	<0.001
0% < vPRA ≤50%	47 (7.1)	39 (6.6)	61 (9.8)	62 (11.0)	84 (15.7)	<0.001
50% < vPRA ≤85%	61 (9.2)	40 (6.8)	53 (8.5)	52 (9.2)	51 (9.6)	0.457
85% < vPRA ≤95%	13 (2.0)	26 (4.4)	27 (4.3)	20 (3.6)	16 (3.0)	0.092
vPRA >95%	11 (1.7)	43 (7.3)	57 (9.2)	60 (10.7)	59 (11.0)	<0.001
vPRA [%], median (IQR)^b^	60.4 (34.1–82.3)	81.5 (46.5–97.6)	73.5 (27.9–96.3)	72.7 (32.1–96.7)	64.6 (23.9–97.1)	0.009

Data are shown as n (% of total) unless indicated otherwise.

^a^ Data from GMZTP excluded.

^b^ Only patients with vPRA >0%. vPRA, virtual panel reactivity based on unacceptable antigen mismatches.

**TABLE 2:** Waiting time in years according to vPRA on 01.06.2025 (n = 534).

vPRA category	Waiting time	​
vPRA = 0%	4.2 (2.7–6.3)
0% < vPRA ≤50%	4.8 (3.1–7.3)
50% < vPRA ≤85%	5.9 (3.2–8.4)
85% < vPRA ≤95%	5.9 (3.3–8.6)
vPRA >95%	7.3 (5.1–10.1)	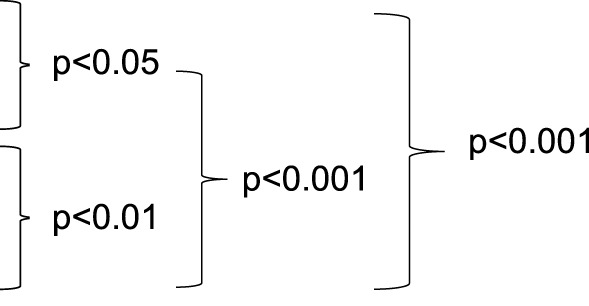

Data are shown as median (IQR).

### Characteristics of Transplanted Patients

267 patients were included in the study, of which 39 (14.6%) had pretransplant DSA with MFI levels below the UAM-SAB criteria. As expected, more DSA-positive patients were sensitized and had higher vPRA levels as compared to DSA-negative patients, with a higher rate of patients with previous transplantations in the former group as compared to the latter. Median MFI^max^ was 2009 (IQR 1373–2988) in DSA-positive patients. The rate of living donations was comparable between the groups (23.1% vs. 21.1%). Thymoglobulin induction was used significantly more often in DSA-positive as compared to DSA-negative patients (61.5% vs. 13.6%). Maintenance immunosuppression consisted of tacrolimus, mycophenolate, and prednisolone in the vast majority of patients. Three patients were lost to follow-up; all other patients were followed for a minimum of 3 years. Median follow-up was 4 years in DSA-positive and 3 years in DSA-negative patients (p = 0.09) (Table 3).

**TABLE 3:** Baseline characteristics of the study cohort.

Characteristic	DSA-positive (n = 39)	DSA-negative (n = 228)	*p*
Transplant center	​	​	0.13
Mainz	15 (38.5)	54 (23.7)	​
Würzburg	2 (5.1)	35 (15.4)	​
Regensburg	18 (46.2)	107 (46.9)	​
Erlangen	4 (10.3)	32 (14.0)	​
Donor			
Female	23 (59.0)	124 (54.4)	0.73
Age [years]	54 (44–59)	56 (47–66)	0.09
Living donor	9 (23.1)	48 (21.1)	​
ETKAS	22 (56.4)	124 (54.4)	​
Full-house allocation	3 (7.7)	22 (9.6)	1.00
ESP	6 (15.4)	50 (21.9)	​
AM	1 (2.6)	1 (0.4)	​
KPTX	1 (2.6)	4 (1.8)	​
HU	0 (0.0)	1 (0.4)	​
HLA-A/B/DR mismatches	4 (3–4)	3 (2–4)	0.14
Transplantation			
Cold ischemia time (h:min)	7:23 (4:27–11:54)	8:05 (4:54–12:18)	0.30
Warm ischemia time (h:min)	0:36 (0:29–0:48)	0:37 (0:30–0:48)^a^	0.49
Recipient			
Female	19 (48.7)	79 (34.6)	0.11
Age [years]	51 (40–62)	57 (47–65)	0.11
HLA antibodies before KT	39 (100.0)	114 (50.0)	<0.001
vPRA >0%	20 (51.3)	27 (11.8)	<0.001
vPRA^b^	71.7 (37.8–89.3)	47.0 (22.0–84.0)	0.16
Retransplantation	12 (30.8)	17 (7.5)	<0.001
Time on dialysis (years)^c^	7.0 (2.8–9.6)	6.4 (3.6–8.7)	0.92
Preemptive	2 (5.1)	16 (7.0)	1.00
AB0-incompatible	2 (5.1)	16 (7.0)	1.00
HLA-DSA			
No. Of HLA-DSA^d^	1 (1–5)	-	-
Class I only	18 (46.2)	-	-
Class II only	19 (48.7)	-	-
Class I + II	2 (5.1)	-	-
MFI^max^	2009 (1373–2988)	-	-
Induction therapy	​	​	<0.001
Basiliximab	15 (38.5)	197 (86.4)	​
Thymoglobulin	24 (61.5)	31 (13.6)	​
Initial immunosuppression	​	​	0.29
TAC-MMF	0 (0.0)	14 (6.1)	​
TAC-MMF-Pred	38 (97.4)	204 (89.5)	​
Other	1 (2.6)	10 (4.4)	​
Follow-up (years)	4.0 (3.0–4.3)	3.0 (3.0–4.0)	0.09

Data are shown as median (IQR) or n (% of total) unless indicated otherwise.

^a^ 2 missing.

^b^ Only vPRA >0%.

^c^ Without preemptive KTX.

^d^ Data are shown as median (range). MFI^max^, highest mean fluorescence intensity of all DSA in cases of more than one DSA.

ETKAS, Eurotransplant Kidney allocation system; ESP, Eurotransplant Senior Program; AM, Acceptable Mismatch program; KPTX, kidney-pancreas transplantation; HU, high urgency; vPRA, virtual panel reactivity; TAC, tacrolimus; MMF, mycophenolate; Pred, prednisolone.

### Waiting Time

Median waiting time prior to deceased donor KT was longer in UAM-positive as compared to UAM-negative patients in both the standard kidney allocation system ETKAS (8.6 vs. 7.7 years) and the senior program ESP (5.6 vs. 4.8 years) [26]. However, these differences were not statistically significant (Supplementary Table S3). Of note, in ETKAS, the difference in median waiting time between UAM-positive and UAM-negative patients decreased to 5 months after exclusion of patients prioritized during allocation because of a full-house (serological match in HLA A, B, and DR) organ (Table 4).

**TABLE 4:** Waiting time (years) prior to KTX.

Allocation program	UAM-positive	UAM-negative	*p*
ETKAS	8.7 (7.6–10.0) [n = 33]	8.2 (6.1–10.3) [n = 91]	0.14
ESP	5.6 (4.6–9.6) [n = 6]	4.8 (3.2–7.0) [n = 49]	0.18

Data are shown as median (IQR). Patients in the acceptable mismatch (AM)-program, with high urgency status and after *full house* allocation, were excluded from analysis.

UAM, unacceptable HLA mismatches; ETKAS, Eurotransplant Kidney Allocation System; ESP, Eurotransplant Senior Program.

### Incidence of AMR

We observed a significantly higher incidence of early AMR in patients with preformed DSA as compared to DSA-negative patients. 4/39 DSA-positive patients experienced AMR within the first 6 months after KT as compared to 3/228 DSA-negative patients (10.3% vs. 1.3%, p = 0.01). 2/4 vs. 2/3 of the respective index biopsies were C4d-positive. 2/4 of the AMR episodes in DSA-positive patients were found in protocol biopsies at 3 months in patients with stable graft function. Of note, six additional DSA-negative patient biopsies fulfilled the criteria of DSA-negative C4d-negative microvascular inflammation (MVI), as proposed by the recent Banff 2022 update [27]. Protocol biopsies were only performed in one (GRBTP) out of the four participating centers. However, the incidence of early AMR and MVI episodes was not statistically different between GRBTP and the other centers (Supplementary Table S4). One of the DSA-positive patients with early AMR lost his graft during follow-up due to a combination of AMR and BK nephropathy following ABO-incompatible living KT. The incidence of early T cell-mediated rejection (TCMR) was comparable between the groups (7.7% vs. 11.4%, p = 0.78).

### Incidence of *De Novo* DSA

Post-transplant DSA screening was performed in approximately 80% of patients (Supplementary Table S8). During follow-up, 4/39 (10.9%) of patients with preformed DSA developed additional *de novo* DSA, whereas *de novo* DSA were detected in 16/228 (7%) of patients without DSA at the time of KT (p = 0.51, Supplementary Table S9).

### Graft Function

Graft function (eGFR) remained stable in both patient groups during follow-up but was significantly higher in DSA-positive patients at early time points (Supplementary Table S5). Albuminuria was generally low but highly variable with no significant differences between DSA-positive and DSA-negative patients (Supplementary Table S6).

### Graft Loss and Patient Death

**FIGURE 2:**
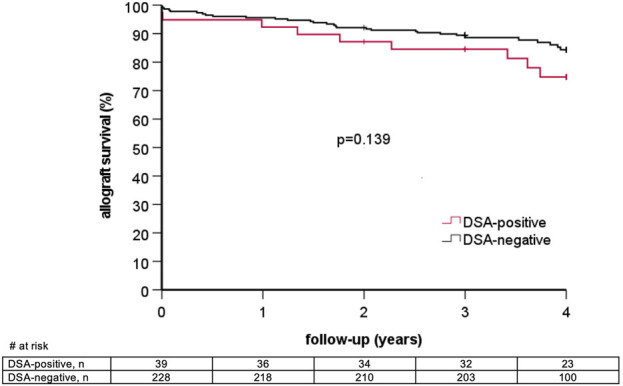
Overall graft survival stratified by the presence of pretransplant donor-specific anti-HLA antibodies (DSA).

**FIGURE 3:**
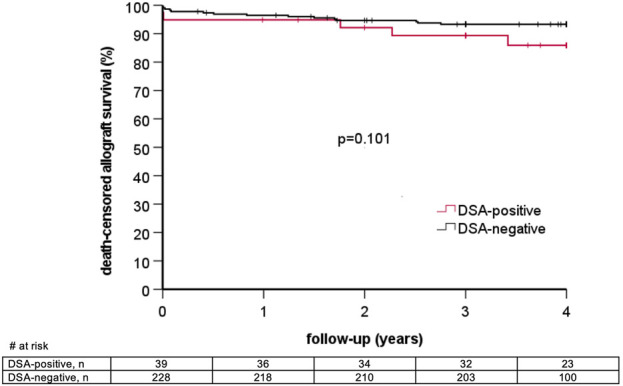
Death-censored allograft survival stratified by the presence of pretransplant donor-specific anti-HLA antibodies (DSA).

6/39 (15.4%) DSA-positive patients lost their graft during follow-up as compared to 16/228 (7.0%) DSA-negative patients (p = 0.11). Graft survival at one, two, and three years in DSA-positive as compared to DSA-negative patients was 92.3% vs. 95.6%, 87.2% vs. 92.1%, and 84.5% vs. 89.5%, respectively (log rank p = 0.14, Figure 2). Two graft losses in the DSA-positive group occurred in patients with previous biopsy-proven AMR. There was no graft loss in patients with previous AMR in the DSA-negative group (Table 5). Graft survival censored for death at one, two, and three years was 94.9% vs. 96.5%, 92.2% vs. 94.7%, and 89.4% vs. 93.3% in DSA-positive vs. DSA-negative patients, respectively (log rank 0.10, Figure 3). Multivariable Cox regression analysis identified thymoglobulin-induction treatment and donor age as independent predictors for graft loss, whereas the presence of preformed DSA and sensitization (vPRA >0%) prior to KT, TCMR, and AMR were not (Table 6).

**TABLE 5:** Graft loss and patient death.

Outcome parameter	DSA-positive (n = 39)	DSA-negative (n = 228)	*p*
Graft loss	6 (15.4)	16 (7.0)	0.11
Graft loss after previous AMR	2 (5.1)	0 (0.0)	0.02
Death	5 (12.8)	22 (9.6)	0.57
Death with functioning allograft	4 (10.3)	16 (7.0)	0.51
Death and/or graft loss	10 (25.6)	32 (14.0)	0.09

Data are shown as n (% of total). DSA, donor specific anti-HLA antibody; AMR, antibody-mediated rejection.

**TABLE 6:** Multivariate Cox regression analysis of graft loss.

Variable	Hazard ratio	95% confidence interval	*p*
Retransplantation	0.260	0.045–1.484	0.13
DSA	2.209	0.673–7.252	0.19
UAM	0.754	0.184–3.091	0.70
Thymoglobulin	4.220	1.560–11.414	0.01
Living donation	0.273	0.060–1.241	0.09
Age of donor	1.047	1.005–1.091	0.03
Age of recipient	0.997	0.959–1.037	0.90
AMR	1.867	0.290–12.038	0.51
TCMR	1.810	0.603–5.438	0.29

DSA, donor specific anti-HLA antibody; UAM, unacceptable HLA mismatches; AMR, antibody-mediated rejection.

During follow-up, 5/39 (12.8%) DSA-positive patients died, whereas death occurred in 22/229 (9.6%) DSA-negative patients (p = 0.57, Table 5). Patient survival at one, two, and three years post KT was comparable between the groups (94.8% vs. 98.2%, 92.2% vs. 94.7% and 92.2% vs. 94.3%, log rank p = 0.70, Supplementary Figure S2). Of note, significantly more DSA-positive patients died from infection as compared to patients without DSA (80.0% vs. 18.2%, p < 0.05, Supplementary Table S7).

## Discussion

A standardized UAM algorithm integrating CDC reactivity, MFI-based SAB test results, and HLA typing information from previous transplants was associated with good short-term outcomes in our cohort. Graft survival of patients with preformed DSA defined as acceptable by the SAB-UAM criteria was superior at 3 years compared to previous studies comprising comparable patient populations and DSA characteristics [8, 10, 12]. At the same time, waiting times between patients with and patients without UAM were not statistically different in both ETKAS and the ESP, underscoring the clinical utility of the chosen UAM criteria.

Given the small sample size of the DSA-positive cohort and the associated low event numbers, we acknowledge that our study is underpowered to demonstrate equivalence in outcome and waiting times between DSA-positive and DSA-negative patients. Likewise, in the Cox model, the number of events relative to the number of covariates was limited, which is why the risk estimates, especially for DSA and UAM, should be interpreted with caution. As protocol biopsies were performed only in one center (GRBTP), and two of the four early AMR episodes in DSA-positive patients were detected on protocol biopsies in patients with stable graft function, there is a potential detection bias in our study (Supplementary Table S4). Again, the low number of events does not justify any final conclusion. Systematic protocol biopsies might be a valuable tool to detect early subclinical rejection in patients with preformed or *de novo* DSA [28, 29], especially as new treatment options for AMR have recently emerged [30].

For the definition of UAM, plausibility testing of SAB test results was restricted to known HLA from previous transplants. To reflect clinical reality, we did not include other sensitizing events such as blood transfusions or previous pregnancies, for which detailed HLA typing information is often not available. Prior transplantations have the strongest impact on allosensitization, likely due to the long-term persistence of alloantigens following KT [31]. However, there is no clinical evidence that antibodies elicited during pregnancies or blood transfusions or even antibodies of unknown etiology are clinically less relevant [32]. In our cohort, the outcome of DSA-positive women with previous pregnancies was not different from all other DSA-positive patients (not shown). Larger studies must be undertaken to find out whether meticulous plausibility testing considering all previous sensitization events can further improve risk stratification.

In case of HLA antibodies not clearly related to a previous KT, we applied MFI cutoffs of 5.000 (10.000 for anti-HLA DQ due to the higher antigen density on anti-DQ beads) for the definition of UAM, as these boundaries were shown to retrospectively identify the majority of DSA-positive KT patients with poor renal outcome [19]. However, it is well established that the MFI only incompletely reflects the immunological risk of a given antibody. Despite a positive correlation of the MFI with early AMR episodes in many studies [7, 8, 10, 14–16], the impact of the MFI on long-term graft survival in DSA-positive patients is less clear [7, 8, 10, 12, 14, 17]. It remains to be shown whether incorporation of dilution/titration studies to address the technical limitations of the SAB assay [33] or incorporation of other test systems, such as B cell memory [34, 35] or C1q [36] assays, will further improve UAM algorithms.

In our study, SAB assays from two different manufacturers were used for risk stratification at the participating tissue typing laboratories. It was previously shown that both assays detect most antibodies with MFI levels above 4000 [37]. However, methodological differences in MFI levels might have consequences on both outcome and waiting times as well when strict MFI thresholds are used for classification of DSA and UAM.

One of the major limitations of our study is the assignment of both SAB-UAM and DSA based on serological HLA typing data. Recently, Senev and colleagues showed that DSA assignment based on second-field high-resolution HLA typing revealed misclassification of donor-specificity in over 20% of patients. This approach was clinically relevant as graft survival in these patients was comparable to DSA-negative patients [38]. High-resolution typing, however, is still not routinely performed at the time of organ allocation in deceased-donor transplantation due to both time and financial constraints but might become available soon [39]. As noted in a recent review by Bezstarosti et al., clinical evidence for a clear benefit for prospective epitope/eplet matching both in terms of waiting time and clinical outcome is still lacking [40]. Nevertheless, allel-specific and molecular assignment of UAM based on epitope/eplet analysis has the potential to further improve individual risk stratification and help enlarge the donor pool, especially in highly sensitized patients. Comparing epitope/eplet patterns of antibody profiles with previous sensitizing events could help establish plausibility when defining UAM [41] and allowed for the delisting of irrelevant UAM in a recent study [42].

Irrespective of how UAM are defined, it is well established that an increasing donor pool restriction results in longer waiting times, with the most dramatic effect in highly sensitized patients [4, 6, 43]. What has not been reported in detail previously is the significant and continuous accumulation of highly sensitized patients on the waiting list following implementation of SAB-UAM. Due to the stringent entry criteria, these patients were not accepted in the ET AM program despite high vPRA levels and a highly restricted donor pool. We have previously shown that the transplant rate of highly sensitized patients not listed in the AM program is less than half than that of AM patients, with this population being numerically twice that of the AM population in Germany [4]. From an equal opportunity perspective, these findings illustrate the urgent need to implement better compensation mechanisms for highly sensitized patients during allocation. Besides potential new therapeutic strategies such as imlifidase induction treatment [44], novel delisting strategies will have to be developed to enable timely transplantation of highly sensitized patients at acceptable immunological risks [36, 45].

Ultimately, sensitization is only one of many factors that influence waiting time prior to KT [4]. Finding the sweet spot between an acceptable immunological risk and increased waiting times remains a critical challenge when defining UAM algorithms. A satisfactory answer to what acceptable waiting times are is highly complex and beyond the scope of this manuscript. Besides the medical aspects that are often discussed in isolation, i.e. the clinical condition of an individual patient and the well-known survival benefit and better quality of life after KT as compared to remaining on dialysis, other aspects such as equity have to be considered as well.

## Data Availability

Publicly available clinical datasets were analyzed for this study and entered into and retrieved from a RedCap-based study database.
