# Supplementary material for: A Prospective Multicenter Luminex-Based Clinical Algorithm to Define Unacceptable HLA Mismatches Before Kidney Transplantation. Consequences on Outcome, Waiting Time, and Wait List Composition
Source: Transpl Int. 2026 Jan 14;38:15497. doi: 10.3389/ti.2025.15497 (PMC12866896; doi:10.3389/ti.2025.15497)

**Supporting Information**

Table S1. Completeness of donor and recipient HLA typing

| **HLA** | **Donor (n=271)** | **Recipient (n=271)** |
| --- | --- | --- |
| A, n (%) | 271 (100.0) | 271 (100.0) |
| B, n (%) | 271 (100.0) | 271 (100.0) |
| C, n (%) | 266 (98.2) | 237 (87.5) |
| DR, n (%) | 271 (100.0) | 271 (100.0) |
| DQA, n (%) | 86 (68.3) | 181 (66.8) |
| DQB, n (%) | 259 (95.6) | 246 (90.8) |
| DPA, n (%) | 168 (62.0) | 139 (51.3) |
| DPB, n (%) | 178 (65.7) | 151 (55.7) |

Table S2. Characteristics of patients on the waiting list over time

|  | 09/2018  n=666 | 06/2019*  n=590 | 03/2022  n=622 | 05/2023  n=563 | 06/2025  n=534 | P |
| --- | --- | --- | --- | --- | --- | --- |
| TX center |  |  |  |  |  |  |
| GNBTP | 271 (40.7) | 257 (43.6) | 234 (37.6) | 187 (33.2) | 180 (33.7) |  |
| GRBTP | 156 (23.4) | 175 (29.7) | 173 (27.8) | 152 (27.0) | 157 (29.4) |  |
| GWZTP | 165 (24.8) | 158 (26.8) | 127 (20.4) | 124 (22.0) | 98 (18.4) |  |
| GMZTP | 74 (11.1) | 0 (0.0)* | 88 (14.1) | 100 (17.8) | 99 (18.5) |  |
| Previous KT |  |  |  |  |  | 0.95 |
| None | 557 (83.6) | 493 (83.6) | 518 (83.3) | 481 (85.4) | 447 (83.7) |  |
| 1x | 96 (14.4) | 83 (14.1) | 86 (13.8) | 71 (12.6) | 70 (13.1) |  |
| 2x | 11 (1.7) | 12 (2.0) | 15 (2.4) | 10 (1.8) | 13 (2.4) |  |
| 3x | 2 (0.3) | 2 (0.3) | 3 (0.5) | 1 (0.2) | 4 (0.7) |  |
| Waiting time [years]** | 4.8  (3.0 - 7.1) | 5.0  (3.0 - 7.3) | 4.8  (3.0 - 7.1) | 5.0  (3.1 - 6.9) | 4.9  (3.0 - 7.2) | 0.87 |

Data are shown as n (% of total) or ** as median (IQR). *Data from GMZTP excluded

Table S3. Waiting time prior to KTX

|  | UAM-positive | UAM-negative | *P* |
| --- | --- | --- | --- |
| ETKAS* | 8.6 (7.2 - 9.9)  n=36 | 7.7 (5.2 - 9.8)  n=110 | 0.08 |
| ESP | 5.6 (4.6 - 9.6)  n=6 | 4.8 (3.2 - 7.0)  n=49 | 0.18 |

* including patients after full-house allocation. Patients in the AM program and with HU status excluded. Waiting time shown in years as median (IQR).

Table S4. Incidence of biopsy-confirmed AMR and MVI (DSA-negative, C4d-negative) episodes at the participating transplant centers divided by protocol vs. indication biopsy

|  | **GRBTP (n=125^#^)** | **GMZTP, GWZTP, GNBTP (n=142^#^)*** | **p** |
| --- | --- | --- | --- |
| **AMR first 6 months**, n (%) | 4 (3.2) | 3 (2.1) | 0.709 |
| protocol vs. indication biopsy | 2/2 | 0/3 |  |
| **MVI (DSA-negative, C4d-negative) first 6 months**, n (%) | 4 (3.2) | 2 (1.4) | 0.423 |
| protocol vs. indication biopsy | 1/4 | 0/2 |  |

^#^number of patients, *at these centers, only indication biopsies were performed.

Table S5. eGFR in DSA-positive vs. DSA-negative patients

| eGFR (CKD-EPI) [ml/min] | DSA-positive (n=39) | DSA-negative (n=228) | *P* |
| --- | --- | --- | --- |
| at 3 months | 52 (36 - 63)  n=37 | 45 (34 - 56)  n=222 | 0.09 |
| at 6 months | 52 (37 - 67)  n=37 | 45 (33 - 56)  n=214 | 0.03 |
| at 12 months | 58 (39 - 74)  n=36 | 48 (35 - 57)  n=217 | 0.03 |
| at 24 months | 57 (35 - 76)  n=34 | 48 (37 - 58)  n=207 | <0.05 |
| at 36 months | 60 (47 - 74)  n=31 | 46 (35 - 58)  n=199 | 0.01 |
| at 48 months | 50 (24 - 69)  n=23 | 49 (35 - 59)  n=98 | 0.87 |
| at 60 months | 56 (27 - 65)  n=7 | 49 (40 - 58)  n=30 | 0.69 |

Data are shown as median (IQR)

Table S6. Albuminuria in DSA-positive vs. DSA-negative patients

| Albuminuria  (mg/g creatinine) | DSA-positive (n=39) | DSA-negative (n=228) | *P* |
| --- | --- | --- | --- |
| at 3 months | 20 (0 - 60.0)  n=35 | 33 (11.0 - 70.0)  n=211 | 0.14 |
| at 6 months | 18 (0 - 37.0)  n=35 | 20.5 (0 - 68.8)  n=200 | 0.1 |
| at 12 months | 13 (0 - 48.0)  n=33 | 23 (0 - 75.0)  n=198 | 0.15 |
| at 24 months | 16 (0 - 92.5)  n=33 | 18 (0 - 70.8)  n=192 | 0.89 |
| at 36 months | 16.5 (0 - 132.5)  n=28 | 20 (0 – 63.0)  n=189 | 0.98 |
| at 48 months | 19.5 (0 - 70.8)  n=22 | 20 (0 – 60.0)  n=95 | 0.69 |
| at 60 months | 40 (0 – 105.0)  n=7 | 0 (0 - 22.5)  n=29 | 0.24 |

Data are shown as median (IQR)

Table S7. Reasons for death

| Reason for death | DSA-positive  (n=5) | DSA-negative  (n=22) | *P* |
| --- | --- | --- | --- |
| Infection | 4 (80.0) | 4 (18.2) | 0.02 |
| Cancer | 1 (20.0) | 2 (9.1) | 0.47 |
| Cardiovascular | 0 (0.0) | 7 (31.8) | 0.28 |
| Unknown | 0 (0.0) | 6 (27.3) | 0.56 |
| Other | 0 (0.0) | 3 (13.6) | 1.00 |

Data are shown as n (%)

Table S8. Completeness of DSA screening post KT

|  | DSA-positive | DSA-negative | p |
| --- | --- | --- | --- |
| 14 days | 31 (79.5)  n=39 | 159 (70.4)  n=226  [missing n=2] | 0.34 |
| 3 months | 32 (86.5)  n=37 | 190 (85.6)  n=222  [missing n=1] | 1.00 |
| 12 months | 31 (86.1)  n=36 | 172 (79.6)  n=216  [missing n=2] | 0.50 |
| 24 months | 28 (82.4)  n=34 | 171 (82.6)  n=207  [missing n=3] | 1.00 |
| 36 months | 23 (74.2)  n=31  [missing n=1] | 134 (66.7)  n=201  [missing n=2] | 0.54 |

Table S9. Incidence of de novo DSA overall

|  | Incidence de novo DSA (overall) | p |
| --- | --- | --- |
| DSA-positive, % | 10.3 (4/39) | 0.51 |
| DSA-negative, % | 7.0 (16/228) |  |

**Figure S1. Patient selection**

**Figure S2. Patient survival**


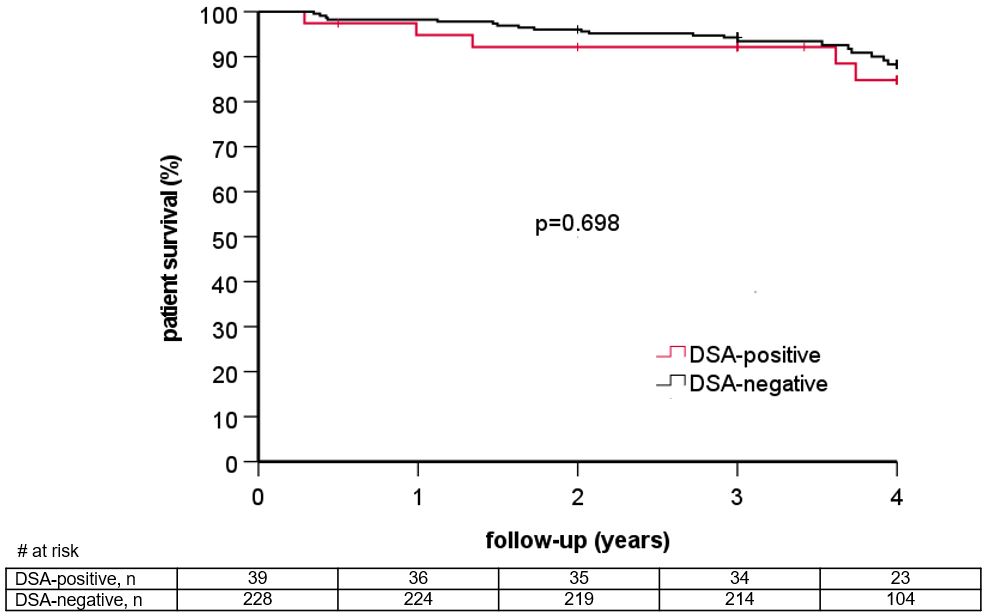

Supplement: Supplementary file 1 [file Supplementaryfile1.docx]
